# *FMR1*: A Neurodevelopmental Factor Regulating Cell Metabolism in the Tumor Microenvironment

**DOI:** 10.3390/biom15060779

**Published:** 2025-05-28

**Authors:** Renbin Zhou, Hao Lin, Xinyu Dou, Bang Zeng, Xinyi Zhao, Lei Ma, Drissa Diarra, Bing Liu, Wei-Wei Deng, Tianfu Wu

**Affiliations:** 1 State Key Laboratory of Oral & Maxillofacial Reconstruction and Regeneration, Key Laboratory of Oral Biomedicine Ministry of Education, Hubei Key Laboratory of Stomatology, School & Hospital of Stomatology, Wuhan University, 237 Luoyu Road, Hongshan District, Wuhan 430079, China; 2 Department of Oral and Maxillofacial Head Neck Surgery, School & Hospital of Stomatology, Wuhan University, 237 Luoyu Road, Hongshan District, Wuhan 430079, China

**Keywords:** FMRP, metabolism, tumor, immune escape, cancer therapy, tumor microenvironment

## Abstract

The Fragile X Mental Retardation 1 (*FMR1*) gene is well-known for its role in Fragile X syndrome, a neurodevelopmental disorder, but emerging evidence suggests its involvement in regulating cellular metabolism, with implications for cancer biology. *FMR1* encodes the Fragile X mental retardation protein (FMRP), an RNA-binding protein that controls various cellular processes, including translation, synaptic plasticity, and RNA metabolism. Recent studies have uncovered novel links between *FMR1*, metabolic regulation, and tumorigenesis. This review discusses the role of *FMR1* in cellular metabolism and its potential involvement in cancer, focusing on glycolysis, mitochondrial metabolism, lipid metabolism, immune cell metabolism, and tumor immune evasion, and as a potential target to enhance immunotherapy, and highlights future research directions to elucidate its mechanistic roles in cancer.

## 1. Introduction

The Fragile X Mental Retardation 1 (*FMR1*) gene, well-known for its association with Fragile X syndrome (FXS), a neurodevelopmental disorder, has traditionally been studied in the context of the central nervous system [1]. The protein encoded by *FMR1*, Fragile X mental retardation protein (FMRP), is an RNA-binding protein that plays a crucial role in regulating mRNA translation, synaptic plasticity, and neuronal development [2,3]. While the function of FMRP in the nervous system has been extensively characterized, emerging evidence suggests that FMRP also exerts significant effects beyond neurons, particularly in the regulation of cellular metabolism and tumor biology [4].

Recent research has deepened our understanding of *FMR1*′s role in cancer biology, highlighting its involvement in key metabolic pathways that support cancer cell survival, proliferation, and immune modulation within the tumor microenvironment (TME) [5]. It is well established that cancer cells undergo metabolic reprogramming to meet their elevated demands for energy, biosynthetic precursors, and survival in the hostile TME [6]. These cells typically achieve this shift by enhancing glycolysis, altering mitochondrial function, and reprogramming lipid metabolism—hallmarks of malignant transformation [7]. FMRP has now been implicated in several of these processes, suggesting it may be a pivotal regulator of cellular development and metabolic adaptation in the context of cancer [8].

The TME, composed of tumor cells, immune cells, endothelial cells, and stromal cells, exhibits remarkable metabolic plasticity that facilitates tumor progression and promotes immune evasion [9]. FMRP’s involvement in regulating the metabolic activities of tumor cells and immune cells within this intricate ecosystem provides new insights into the mechanisms by which tumors modulate immune responses. By influencing immune cell metabolism, FMRP may significantly impact the efficacy of cancer immunotherapies, particularly immune checkpoint blockade therapies, which are increasingly employed to treat a wide range of malignancies.

In this review, we discuss the multifaceted roles that the *FMR1* gene and its product FMRP play in controlling cellular metabolism. We first introduce the involvement of FMRP in key metabolic pathways, such as glycolysis, mitochondrial function, and lipid metabolism, and then outline the impact of FMRP on tumor immunity and immune evasion, with a particular focus on its impact on the cancer microenvironment. As for the possibility of utilizing FMRP as a promising target for cancer therapy, given the central role of FMRP in controlling metabolic pathways, immune cell activity, and tumor progression, targeting FMRP suggests to be a novel approach to enhance antitumor therapy. By focusing on these novel roles of FMRP, we hope to provide a comprehensive overview of how FMRP, traditionally considered a neurodevelopmental factor, is increasingly being recognized as an essential element in cancer metabolism and immunology.

## 2. Methodology

This narrative review aimed to synthesize and discuss the current understanding of FMRP’s role in cellular metabolism and cancer. We selected PubMed, Web of Science, and Scopus for literature searching due to their comprehensive coverage of biomedical and life science research, ensuring a broad and authoritative data source for our topic. The search included studies published between 2000 and 2025, particularly focusing on articles from the past five years. This time frame was chosen to capture the most recent and relevant findings, as research on FMRP’s role in cancer metabolism has rapidly advanced in recent years.

Our search strategy combined Medical Subject Headings (MeSH) terms and keywords. “*FMR1*” OR “FMRP” were used to target studies on the gene and its encoded protein. Terms like “tumor microenvironment” OR “cancer metabolism”, “glycolysis” OR “mitochondrial metabolism” OR “lipid metabolism”, “immune evasion” OR “tumor immunity”, and “cancer therapy” OR “therapeutic targeting” were incorporated to focus on the relevant aspects of cancer biology. Boolean operators (AND and OR) refined the search, ensuring that only studies closely related to FMRP’s role in tumor metabolism and immune regulation were retrieved. We also limited the search to peer-reviewed articles in English to maintain the quality and relevance of the literature.

We defined strict inclusion and exclusion criteria. Eligible articles included peer-reviewed original research and narrative or systematic reviews, specifically those examining FMRP’s role in cancer metabolism, the tumor microenvironment, or therapeutic applications. We prioritized in vitro, in vivo, and clinical studies on FMRP-related pathways, such as its impact on cancer cell glycolysis or tumor immune cell infiltration. Conversely, we excluded conference abstracts, non-peer-reviewed works (e.g., unreviewed preprints), and studies irrelevant to cancer biology, like those focusing solely on FMRP’s role in neurodegenerative diseases.

We reviewed the full texts of potentially relevant articles for eligibility. Finally, we categorized the selected articles by FMRP’s molecular mechanisms, its roles in metabolic reprogramming and immune modulation, and its therapeutic potential to synthesize the current knowledge systematically.

## 3. *FMR1* and FMRP: Beyond Neurodevelopment

FMRP’s capacity to regulate multiple aspects of cellular metabolism suggests that it is essential for maintaining cellular homeostasis under normal and disease conditions [10]. By controlling key metabolic pathways that sustain cell growth, survival, and energy production, FMRP emerges as a pivotal factor in normal cellular function and cancer biology [11]. Its expression across diverse cell types, including cancer cells, immune cells, and stromal cells, underscores its broader involvement in the TME and suggests that FMRP serves as a central regulator of metabolic reprogramming during cancer progression [5].

### 3.1. Function of FMRP in Normal Physiology

**Figure 1:**
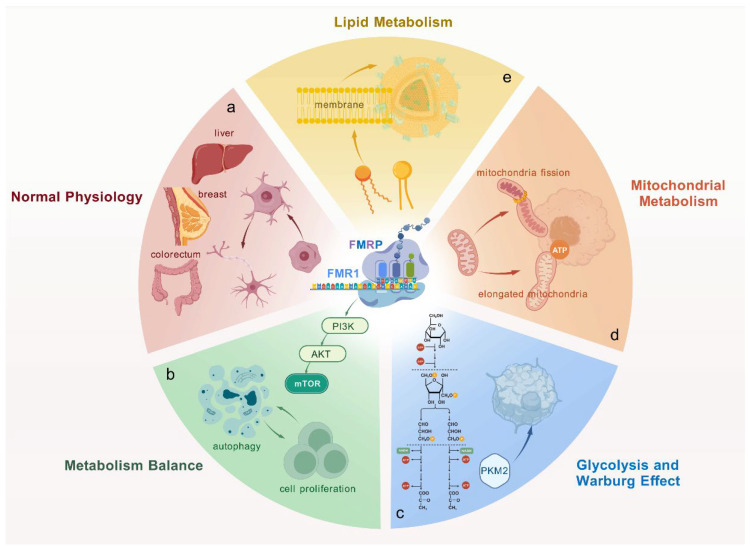
Fragile X mental retardation protein (FMRP): from cellular metabolism to cancer progression. (**a**) FMRP regulates protein synthesis at synapses, which is crucial for neural development and function. It is also involved in non-neuronal tissues such as the breast, colon, and liver. (**b**) FMRP interacts with the PI3K/AKT/mTOR pathway, central to cellular metabolism and growth, suggesting a role in cancer cell metabolism. (**c**) The silencing of *FMR1* promotes the increased expression of key glycolytic enzymes. (**d**) FMRP modulates mitochondrial dynamics, driving mitochondrial fission and elongation, and thereby regulating ATP production. (**e**) FMRP influences lipid metabolism in cancer cells, potentially maintaining energy balance and growth through lipid synthesis and fatty acid oxidation. Created with BioGDP.com [35].

FMRP modulates synaptic protein synthesis by regulating the translation of multiple mRNAs, which influences synapse formation and remodeling. These processes are essential for neural development and function [12] (Figure 1a). While FMRP is best known for its role in the central nervous system (CNS), it is also expressed in various peripheral tissues, including the breast, colon, and liver. Many of these tissues are closely associated with immune responses and oncogenesis [13] (Figure 1a). Although its function in these non-neuronal tissues remain less well characterized, emerging evidence suggests that FMRP’s regulatory role extends beyond the CNS and includes the control of metabolic pathways that are critical for maintaining cellular homeostasis [14].

FMRP interacts with several signaling pathways that govern cell proliferation, survival, and stress responses [15]. In particular, it is involved in the PI3K/AKT/mTOR pathway, which is a major regulator of cellular metabolism and growth [16,17] (Figure 1b). The mammalian target of rapamycin (mTOR) pathway is essential for metabolic reprogramming in cancer, and FMRP’s interaction with this pathway points to its potential role in cancer cell metabolism [18] (Figure 1b). This pathway regulates protein synthesis, cell growth, and autophagy, thereby ensuring metabolic balance in healthy and cancerous cells [19].

### 3.2. FMRP’s Role in Cell Metabolism and Cancer Progression

As our knowledge of FMRP’s function in cancer biology evolves, it is already clear that FMRP regulates key aspects of cellular metabolism, including nutrient uptake, energy production, and stress responses, all of which are critical for the survival of cancer cells. [20]. To support their rapid proliferation and division, cancer cells frequently undergo metabolic reprogramming [21]. FMRP’s regulation of metabolic pathways such as glycolysis, mitochondrial function, and lipid metabolism indicates its crucial contribution to enabling cancer cells to adapt to the metabolic demands of the TME [22,23].

Glycolysis and the Warburg effect: Glycolysis is an oxygen-independent metabolic pathway that converts glucose into pyruvate, occurring in the cytoplasm of most organisms’ cells [24,25]. The upregulation of glycolysis, even in the presence of oxygen and known as the Warburg effect, is a hallmark of many cancers [26]. This metabolic adaptation allows tumor cells to rapidly generate ATP and biosynthetic intermediates required for proliferation [27]. Research has unveiled that the silencing of FMRP triggers a significant upregulation of a series of metabolic enzymes. Specifically, the expression of enzymes in the glycolytic pathway, including hexokinase II (HK2), pyruvate kinase M2 (PKM2), and lactate dehydrogenase A (LDHA) show markedly increased expression, along with elevated intracellular lactate levels. Concurrently, enzymes involved in the tricarboxylic acid (TCA) cycle and NAD+/NADH metabolism, including components of the malate/aspartate shuttle and isocitrate dehydrogenase, are also upregulated [28] (Figure 1c). These metabolic reprogramming alterations underscore the profound regulatory role of FMRP silencing in cellular energy metabolism, offering novel molecular insights into cancer metabolic adaptability.

Mitochondrial metabolism and dynamics: Mitochondria serve as central hubs for energy production and biosynthesis. Their dysfunction is a hallmark of cancer [29]. FMRP regulates mitochondrial dynamics by promoting local translation of mitochondrial fission factor (MFF) mRNA. MFF facilitates mitochondrial fission, a process vital for mitochondrial health, biogenesis, and cellular distribution. FMRP enhances mitochondrial fission at the midzone, optimizing oxidative phosphorylation (OXPHOS). This boosts ATP synthesis and metabolic flexibility in cancer cells [30,31] (Figure 1d). By fine-tuning mitochondrial dynamics, FMRP plays a central role in tumor energy metabolism. Its influence on mitochondrial biogenesis may also increase electron transport chain activity, enhancing energy output under stress conditions. This metabolic adaptability is crucial for cancer cells in nutrient-poor environments, supporting their survival and growth.

Lipid metabolism: FMRP regulates lipid metabolism by inhibiting the translation of key enzymes like *CPT1A* and *SLC16A1* [22]. Its absence increases lipolysis, promotes hepatic fatty acid oxidation (FAO), reduces lipid storage, and increases circulating free fatty acids and ketone bodies [22] (Figure 1e). These metabolic shifts profoundly impact tumor biology. In FAO-dependent cancers, such as pancreatic and prostate cancer, FMRP loss boosts lipid availability, fueling tumor growth and metabolic adaptation [32]. Conversely, in glycolysis-driven tumors such as breast cancer and melanoma, FMRP promotes lipid accumulation and suppresses FAO, supporting rapid proliferation [33]. FMRP-driven lipid regulation enhances immunosuppressive cells like tumor-associated macrophages (TAMs) and myeloid-derived suppressor cells (MDSCs), facilitating immune evasion and tumor progression [34]. By shaping tumor metabolism and the TME, FMRP emerges as a key regulator in cancer development and holds promise as a therapeutic target in lipid-dependent malignancies.

### 3.3. Regulation and Cell-Type-Specific Functions of FMRP

FMRP undergoes tight regulation through multiple mechanisms. Epigenetic modifications, including CGG-repeat expansions in the 5′ untranslated region of the *FMR1* gene, actively modulate its transcription by inducing DNA hypermethylation and transcriptional silencing in FXS [36]. Post-transcriptionally, alternative splicing generates FMRP isoforms with distinct RNA-binding properties. Phosphorylation further regulates FMRP’s association with polyribosomes [37]. FMRP binds mRNAs through its KH domains and RGG box, repressing translation by inhibiting ribosomal elongation or recruiting silencing complexes [38]. Cellular signals also modulate FMRP activity. The metabotropic glutamate receptor (mGluR) pathway upregulates FMRP synthesis via secondary messenger systems, particularly near synapses [39]. This dynamic regulation positions FMRP as a critical mediator of cellular responses to environmental and developmental cues.

FMRP exhibits distinct roles across cell types, reflecting its broad impact on cellular physiology. In neurons, FMRP regulates synaptic plasticity by repressing the translation of mRNAs encoding synaptic scaffolding proteins, such as *Shank3*. This shapes dendritic spine architecture and supports cognitive function. FMRP loss in FXS leads to synaptic dysfunction and intellectual disability [40,41]. In astrocytes, selective FMRP deficiency disrupts GLT1-mediated glutamate uptake. This disrupts glutamate clearance and contributes to cortical synaptic dysfunction in FXS [42]. In tumor cells, such as glioblastoma, FMRP promotes proliferation by stabilizing mRNAs encoding transcription factors of canonical WNT/β-catenin and non-canonical WNT-ERK1/2 signaling pathways, including *β-catenin*, *CREB*, and *ETS1*. FMRP depletion suppresses tumor growth [43]. These cell-type-specific functions highlight FMRP’s versatility and therapeutic potential in neurological, oncological, and immunological disorders.

## 4. FMRP’s Role in the Tumor Microenvironment

The TME is a complex and dynamic ecosystem. It includes tumor cells, immune cells, stromal cells like fibroblasts and endothelial cells, the extracellular matrix (ECM), and blood vessels [44]. This microenvironment significantly influences tumor development, spread, and therapy resistance [45]. Growing evidence shows that *FMR1* and its protein, FMRP, regulate metabolism in various TME cell types. This regulation shapes overall tumor biology [46]. By modulating metabolism in both cancerous and non-cancerous cells, FMRP affects tumor growth, immune responses, and immune evasion strategies within the TME [11].

### 4.1. FMRP and Endothelial Cells in the Tumor Microenvironment

**Figure 2:**
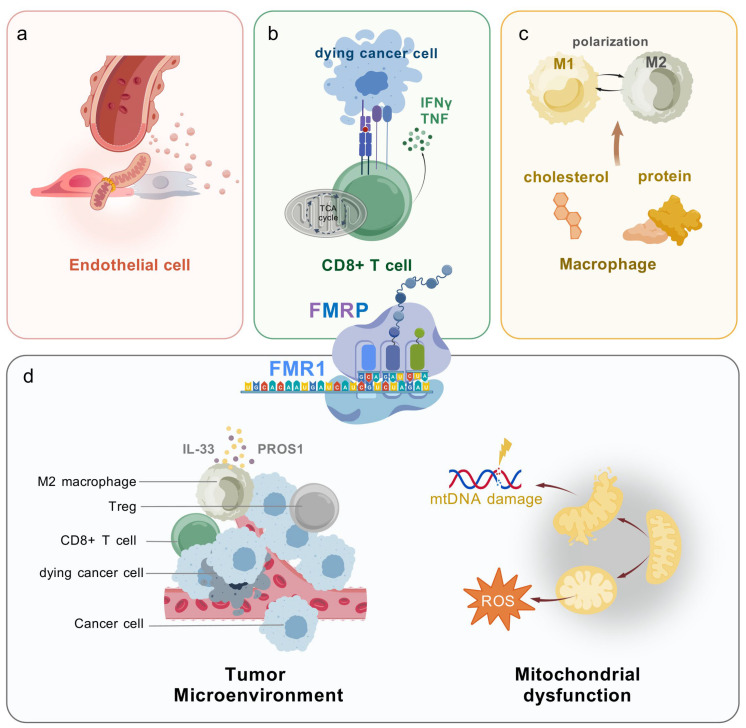
FMRP’s role in metabolic regulation and immune evasion within the tumor microenvironment (TME). (**a**) FMRP enhances mitochondrial translation, potentially indirectly influencing endothelial cell angiogenic function. (**b**) FMRP modulates glycolytic enzymes to regulate T cell activation and cytokine release, including Interferon-gamma (IFNγ) and Tumor Necrosis Factor (TNF), shaping their ability to target tumor cells effectively. (**c**) FMRP induces cholesterol metabolism abnormalities and protein misfolding, thereby influencing the polarization of M1 and M2 macrophages. (**d**) FMRP promotes the formation of M2 macrophages by modulating the expression of immunoregulatory factors such as Interleukin-33 (IL-33) and Protein S (PROS1), while reducing CD8(+) T-cell infiltration and increasing Treg infiltration, thereby fostering an immunosuppressive TME. Additionally, FMRP induces mitochondrial DNA damage, impairing mitochondrial function and oxidative stress regulation, leading to elevated reactive oxygen species (ROS) levels and subsequent immune dysfunction. Created with BioGDP.com [35].

In response to pathological angiogenesis, endothelial cells swiftly transition from a quiescent state to a state of proliferation and migration, with their unique glycolytic characteristics playing a crucial role in these processes [47,48]. The Fang research team discovered that FMRP regulates angiogenesis via the miR-181a-CaM-CaMKII signaling pathway. FMRP deficiency upregulates miR-181a, which suppresses calmodulin (CaM) and CaMKII signaling. This impairs endothelial cell proliferation and tube formation. The pro-inflammatory cytokine TNF-α exacerbates this by inducing FMRP dephosphorylation, enhancing miR-181a biogenesis, and inhibiting CaM-CaMKII activity. Conversely, overexpressing constitutively phosphorylated FMRP (FMRP-S500D) reverses TNF-α-mediated suppression of endothelial proliferation and angiogenesis [49]. Emerging evidence demonstrates that silicon modulates mitochondrial fission dynamics in macrophages via the Drp1-Mff pathway and facilitates the transfer of functional mitochondria to endothelial and neuronal cells through microvesicle-mediated intercellular transport, thereby promoting angiogenesis and neurodevelopment [50]. This finding provides novel experimental support for the regulatory role of the FMRP-Mff axis in endothelial cell function (Figure 2a). Through these mechanisms, FMRP emerges as a critical regulator of endothelial cell metabolism and angiogenesis.

### 4.2. FMRP’s Impact on Immune Cells and Tumor Immunity

The immune system is critical for recognizing and eliminating tumor cells. However, tumors often develop strategies to evade immune detection [51]. Metabolic alterations in immune cells are key to immune evasion. Tumor cells alter immune cell metabolism to reduce their effectiveness [52]. FMRP’s regulation of immune cell metabolism is an emerging area of interest, as it may positively and negatively influence immune responses within the TME.

The immune effects of T cell glycolysis: T cells, especially cytotoxic T lymphocytes (CTLs), are vital for antitumor immune responses [53]. However, their ability to function effectively depends on their metabolic state [54]. FMRP regulates T cell glycolysis, a key factor in their activation and differentiation [55]. Glycolysis provides energy for rapid division and function in activated T cells [56]. FMRP has been demonstrated to engage with the mTORC1 signaling cascade, a major regulator of T cell metabolic reprogramming [57,58] (Figure 2b). Specifically, FMRP modulates mTORC1 activity by regulating the translation of upstream components like *LAMTOR1*, which is essential for amino acid sensing and mTORC1 activation [49]. Additionally, mTORC1 may phosphorylate FMRP, indicating a bidirectional regulatory relationship that integrates nutrient signaling with T cell metabolic adaptation [59]. This interplay underscores FMRP’s dual role as both a regulator and an effector within the mTORC1 pathway, highlighting its importance in metabolic signaling and immune function. By enhancing mTORC1 activity, FMRP promotes the activity of genes associated with glycolysis, increasing the glycolytic capacity of T cells. However, within the TME, high FMRP expression, potentially driven by tumor-derived signals or chronic antigen stimulation, may shift its role from promoting glycolysis to inducing metabolic suppression, contributing to T cell exhaustion. In contrast, during early activation stages, FMRP supports glycolytic reprogramming via mTORC1 [5]. This dual effect indicates that FMRP’s role in T cell metabolism is context-dependent, varying across different cell types and microenvironmental conditions.

Macrophages and immune polarization: Macrophages are a key component of the TME and exhibit distinct phenotypic shifts in response to metabolic cues [60]. Pro-inflammatory M1 macrophages suppress tumors and rely primarily on glycolysis. In contrast, immunosuppressive M2 macrophages promote tumor progression and prefer FAO for energy [61,62]. Recent studies suggest that FMRP might contribute to controlling macrophage metabolism, given its demonstrated impact on lipid and glucose balance [22]. Loss of FMRP has been associated with disrupted lipid metabolism, including reduced circulating lipid levels, such as cholesterol, as observed in *FMR1*-knockout (*FMR1*-KO) mice and individuals with FXS [63] (Figure 2c). Since TAMs heavily rely on scavenger receptor *CD36* for lipid uptake, it has been hypothesized that FMRP deficiency may reduce lipid availability, potentially altering macrophage metabolic programming [64]. However, direct experimental evidence linking FMRP deficiency to *CD36*-mediated lipid uptake in TAMs remains limited, and further studies are needed to confirm this relationship. In addition, lipopolysaccharide (LPS)-activated M1 macrophages exhibit high glycolytic activity, a hallmark of the Warburg effect, which supports their inflammatory cytokine production and bactericidal function [65,66]. Conversely, M2 macrophages express high levels of *CARKL*, an enzyme that reduces pentose phosphate pathway (PPP) activity. This leads to lower glutathione (GSH) levels and reduced inflammatory cytokine production [67]. Given FMRP’s role in metabolic regulation, it likely influences the balance between M1 and M2 macrophage polarization by modulating lipid and glucose metabolism. However, the precise mechanisms by which FMRP affects macrophage function remain unclear, and further experimental studies are required to establish direct links between FMRP and immune polarization in the TME.

### 4.3. Tumor Immune Evasion and FMRP’s Role

Cancer cells have evolved strategies to escape immune detection, including altering immune cell metabolism, suppressing immune cell function, and hijacking the immune system to promote tumor growth [68]. One key immune evasion strategy employed by tumors is creating an immunosuppressive TME, driven by metabolic factors [69]. FMRP’s role in regulating metabolic processes in cancer and immune cells suggests that it may contribute to immune evasion through multiple mechanisms.

Regulating immune factors: FMRP plays a key role in tumor immune evasion. It influences the interaction between cancer and the immune system through various metabolic pathways and immune regulatory mechanisms [5]. Specifically, FMRP encourages the development of immunosuppressive M2 macrophages by modulating the activity of immune-regulating factors like Interleukin-33 (IL-33) and Protein S (PROS1) while inhibiting the production of the pro-inflammatory molecule C-C Motif Chemokine Ligand 7 (CCL7), thereby decreasing CD8(+) T cell infiltration and boosting regulatory T cells (Tregs)’ presence [5,70,71] (Figure 2d). Furthermore, the loss of FMRP reshapes the TME, leading to T lymphocyte activation, particularly CD8(+) T cells, thereby enhancing their tumor-killing capacity [5].

ROS and immune dysfunction: Elevated reactive oxygen species (ROS) levels in the TME cause immune dysfunction. FMRP regulates oxidative stress and mitochondrial activity. It may control ROS levels, impacting the immune system’s ability to mount an effective antitumor response [72,73] (Figure 2d). In colorectal cancer cells, FMRP expression correlates with ROS regulation, oxidative stress-induced cell death, and mitochondrial respiration, encompassing a range of critical biological processes [74]. By modulating ROS in tumor and immune cells, FMRP may help tumors evade immune detection.

## 5. FMRP as a Target for Cancer Therapy

FMRP plays a growing role in regulating cellular metabolism and immune responses within the TME. This highlights its potential as a novel therapeutic target for cancer treatment. FMRP influences metabolic reprogramming, immune modulation, and tumor progression. Targeting this protein could enhance existing therapies, including chemotherapy, immunotherapy, and targeted treatments. In this section, we explore the therapeutic implications of modulating FMRP activity, the associated challenges, and the potential of FMRP as a therapeutic target.

### 5.1. FMRP’s Role in Immunotherapy and Immune Modulation

Immunotherapy has shown success in cancers like melanoma and lung cancer. This highlights the potential of immune-based therapies [75]. However, the TME significantly hinders broader efficacy [76,77]. FMRP is a potential target in cancer immunotherapy because it regulates immune cell metabolism and modulates the TME [78]. Mechanistically, FMRP suppresses CTL function by inhibiting glycolysis-related enzymes, leading to metabolic exhaustion of tumor-infiltrating T cells [79]. FMRP also promotes the polarization of TAMs toward an M2-like state, aiding immune evasion by enhancing the release of Interleukin-10 (IL-10) and Transforming Growth Factor-beta (TGF-β) [5]. These impacts establish FMRP as a hopeful target for addressing resistance mechanisms in treatments such as anti-PD-1, anti-PD-L1, and anti-CTLA-4 therapies, which often fail due to metabolic exhaustion or the accumulation of immunosuppressive cells like Tregs [80].

Therapeutic strategies targeting FMRP could reverse immune suppression and restore T cell function. Small-molecule inhibitors targeting FMRP’s RNA-binding domain may enhance T cell metabolism and sensitize tumors to immune checkpoint inhibitors (ICIs) [81]. Furthermore, FMRP-specific proteolysis-targeting chimera (PROTAC) degraders have demonstrated potential in preclinical models, enhancing immune cell infiltration and tumor clearance [79].

### 5.2. Combination Therapies Targeting FMRP

Given the extensive role of FMRP in tumor metabolism and immune suppression, inhibiting FMRP alone may not suffice as a monotherapy. Instead, combining FMRP-targeted strategies with metabolic and immunotherapeutic agents could enhance therapeutic efficacy and overcome resistance mechanisms. FMRP inhibition alters cancer cell adaptation by modulating gene expression tied to metabolic and immune regulation. Since FMRP regulates glycolysis and OXPHOS, combining its inhibition with glycolytic blockers such as 2-deoxyglucose (2-DG) or HK2 inhibitors could impair tumor energy metabolism and sensitize tumors to therapy. Metformin, an OXPHOS inhibitor, has synergized with FMRP-targeting strategies. This combination could starve tumor cells and reduce treatment resistance [82,83]. FMRP’s immunosuppressive role suggests that its inhibition could enhance immune checkpoint blockade therapy. Tumors with high FMRP expression exhibit poor responses to ICIs due to metabolic exhaustion of cytotoxic T cells [81]. Combining FMRP inhibition with ICIs could restore T cell metabolic fitness, leading to a more effective antitumor response. FMRP also supports cancer stem cell (CSC) maintenance by supporting lipid metabolism. This process is vital for CSC survival, therapy resistance, and tumor relapse [84]. Inhibiting FMRP alongside fatty acid synthase (*FASN*) inhibitors may deplete CSC populations, improving long-term treatment outcomes [85]. By integrating FMRP-targeted therapies with metabolic inhibitors, immune checkpoint blockade, and CSC-directed approaches, a comprehensive strategy to overcome tumor progression and resistance can be developed.

### 5.3. Challenges in Targeting FMRP in Cancer Therapy

Targeting FMRP as a therapeutic strategy holds significant potential. However, several challenges must be addressed to ensure its clinical viability. Specificity is a major concern. FMRP is essential for neuronal development. Systemic inhibition may cause off-target effects and neurotoxicity [86]. Developing highly selective modulators is critical. These should target FMRP’s oncogenic roles in cancer cells while preserving its functions in neurons. This approach minimizes adverse effects. Effective delivery systems also pose a challenge. Advanced technologies, such as nanoparticle-based drug carriers or CRISPR/Cas9 gene editing, could improve targeted delivery to tumor cells. This would spare healthy tissues and enhance therapeutic efficacy and safety [87]. Resistance mechanisms are another limitation [88]. Tumors may adapt to FMRP inhibition through alternative metabolic or signaling pathways, necessitating combination strategies that simultaneously target FMRP and other critical pathways within the TME [89]. These integrative approaches may help overcome resistance and sustain durable therapeutic responses. Addressing these challenges through innovative drug design, delivery systems, and combination therapies will be crucial to realizing the full potential of FMRP-targeted cancer treatments.

## 6. FMRP and Cancer Metabolism in Specific Cancer Types

FMRP regulates cellular metabolism and immune modulation in the TME. It influences cancer progression across various malignancies. Across various cancer types, FMRP commonly regulates glycolysis, mitochondrial function, lipid metabolism, and immune evasion, supporting tumor proliferation and survival. However, its specific mechanisms and molecular targets vary by cancer type, reflecting the unique metabolic and microenvironmental demands of each malignancy. This section summarizes the shared roles of FMRP in cancer metabolism and compares its distinct contributions in breast cancer, glioblastoma (GBM), intrahepatic cholangiocarcinoma (iCCA), and head and neck squamous cell carcinoma (HNSCC), highlighting cancer-specific differences.

### 6.1. Common Roles of FMRP in Cancer Metabolism

FMRP plays a consistent role in cancer progression. It regulates metabolic and immune processes. These enable tumor cells to adapt to challenging microenvironments. FMRP enhances glucose metabolism to support the rapid energy production and biosynthetic demands essential for tumor growth [22,90,91]. It modulates mitochondrial activity and lipid metabolism, contributing to the energy homeostasis and structural requirements for cell proliferation and survival [92,93]. Furthermore, FMRP promotes immune evasion by fostering an immunosuppressive tumor microenvironment, limiting immune cell activity and enhancing tumor persistence [94]. These shared functions underscore FMRP’s ability to sustain tumor adaptation across various cancers. However, its specific molecular targets and regulatory mechanisms differ by cancer type, as detailed in the following sections.

### 6.2. Cancer-Specific Roles of FMRP

#### 6.2.1. Breast Cancer

In breast cancer, FMRP catalyzes N6-methyladenosine (m6A) modification of Solute Carrier Family 7 Member 11 (*SLC7A11*) mRNA and interacts with Heterogeneous Nuclear Ribonucleoprotein M (hnRNPM) to mediate *SLC7A11*-S splicing, promoting ferroptosis resistance [95]. It may contribute to aerobic glycolysis, potentially supporting tumor proliferation by stabilizing metabolic pathways critical for energy production [96]. FMRP also enhances O-GlcNAcylation via the hexosamine biosynthesis pathway (HBP), upregulating Topoisomerase II Alpha (TOP2A) activity to drive cell cycle progression [97]. These diverse roles position FMRP as a potential therapeutic target for disrupting breast cancer progression.

#### 6.2.2. FMRP in Glioblastoma

In glioblastoma, increased FMRP expression is associated with poor patient prognosis, while its downregulation inhibits tumor growth. FMRP enhances WNT/β-catenin signaling, promoting the proliferation of Glioblastoma Stem Cells (GSC) [43]. FMRP regulates glycolysis, a key process that enables GBM cells to adapt to hypoxic environments and rapidly acquire energy [28,98]. Glycolysis facilitates GBM immune evasion through HK2-mediated phosphorylation of the Inhibitor of Nuclear Factor Kappa B Alpha (IκBα). [99]. Notably, this role of FMRP in promoting glycolysis in glioblastoma contrasts with findings in other contexts, where silencing FMRP upregulates glycolytic enzymes, suggesting that FMRP normally suppresses glycolysis [28]. This apparent contradiction highlights FMRP’s context-dependent regulation of metabolic pathways, which may vary between tumor types or cellular contexts, such as tumor cells versus immune cells, as discussed in Section 4.2. These findings offer insights into how FMRP may influence GBM progression through glycolytic regulation.

#### 6.2.3. FMRP in Intrahepatic Cholangiocarcinoma

FMRP contributes to metastatic progression in iCCA. This hepatobiliary malignancy has limited treatment options. FMRP regulates mRNA networks that drive cytoskeletal remodeling and ECM degradation—key processes in tumor invasion and dissemination [100]. FMRP promotes tumor invasion by stabilizing mRNAs of invasion-related proteins, including Cortactin (*CTTN*), Matrix Metalloproteinase 1 and 9 (*MMP1/9*), and Proto-oncogene Tyrosine-protein Kinase Src (*SRC*) [101]. FMRP knockdown disrupts invadopodia formation. It also reduces gelatinolytic activity and impairs tumor cell migration and invasion. These effects are reversed upon FMRP re-expression. FMRP’s enrichment at the tumor–stromal interface suggests its involvement in microenvironmental interactions, potentially amplifying pro-invasive signaling pathways [100]. Given these findings, targeting FMRP’s RNA-binding activity or its downstream effectors, such as Cortactin, may present a promising therapeutic strategy to mitigate iCCA metastasis.

#### 6.2.4. FMRP in Head and Neck Cancer

**Figure 3:**
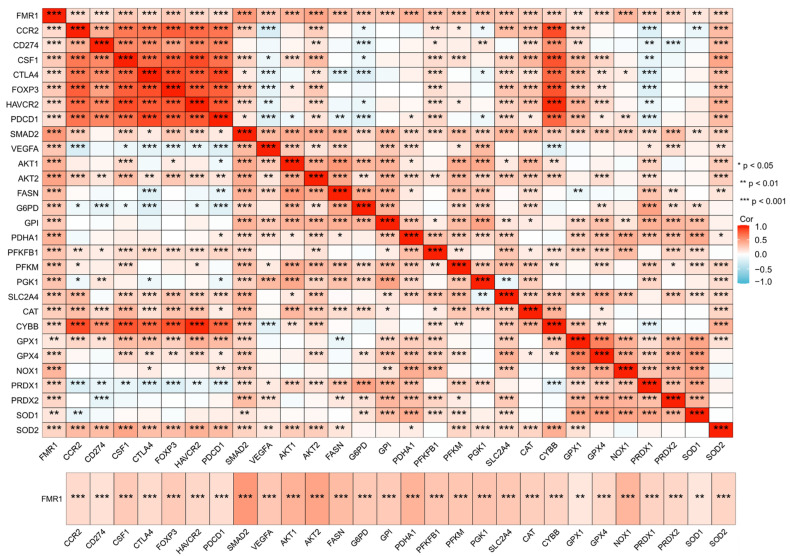
Correlation heatmap of *FMR1* with representative genes in immune evasion, glycolysis, and ROS pathways. The heatmap illustrates the correlation between *FMR1* and genes categorized by functional pathways: *CCR2* to *VEGFA* represents immune evasion, *AKT1* to *SLC2A4* represents glycolysis, and *CAT* to *SOD2* represents the ROS pathway. The color gradient from blue to red indicates correlation strength and direction, with blue representing negative and red representing positive correlations. Significance levels are denoted by *, **, and ***, corresponding to *p*-values < 0.05, <0.01, and <0.001, respectively. Created by Xiantao tool (https://www.xiantao.love/, accessed on 1 April 2025).

There is a notable lack of in-depth research exploring the role of FMRP in HNSCC. However, analysis of The Cancer Genome Atlas (TCGA) database reveals strong correlations. FMRP associates with key factors in glucose metabolism, ROS pathways, and immune evasion. This suggests a role in HNSCC progression (Figure 3). FMRP correlates with glycolytic enzymes, including 6-phosphofructo-2-kinase/fructose-2,6-bisphosphatase 1 (*PFKFB1*), phosphofructokinase muscle type (*PFKM*), and phosphoglycerate kinase 1 (*PGK1*). It also associates with *FASN*. These links indicate FMRP’s role in metabolic reprogramming. This supports tumor proliferation and survival. FMRP also correlates with antioxidant enzymes, such as glutathione peroxidase 4 (*GPX4*) and peroxiredoxin 1 (*PRDX1*). This suggests it maintains redox homeostasis and protects tumor cells from oxidative stress. FMRP’s associations with immune checkpoint molecules—including programmed cell death protein 1 (*PDCD1*), programmed death-ligand 1 (*CD274/PD-L1*), and hepatitis A virus cellular receptor 2 (*HAVCR2/TIM-3*)—highlight its potential involvement in promoting immune evasion within the TME. These findings position FMRP as a potential regulator at the intersection of metabolic pathways and immune modulation, contributing to HNSCC progression. While the precise mechanisms remain unclear, these preliminary findings provide new insights into FMRP’s multifaceted role. They also provide a foundation for future research and targeted therapeutic strategies in HNSCC.

### 6.3. Controversies and Tumor-Specific Mechanisms of FMRP in Cancer Biology

While accumulating evidence supports the pivotal role of FMRP in cancer metabolism, immune evasion, and therapeutic resistance across various malignancies, several controversies and limitations warrant further investigation. Notably, the oncogenic or tumor-suppressive functions of FMRP appear highly context-dependent, varying by cancer type, molecular subtype, and TME. However, a retrospective case-control study of 127 breast cancer patients revealed that lower FMRP levels in primary HER2-positive tumors were associated with increased metastasis, suggesting a potential tumor-suppressive role in specific subtypes [102]. This finding contrasts with FMRP’s oncogenic roles in other breast cancer subtypes, where it promotes ferroptosis resistance and glycolysis [95,96]. The tumor-suppressive role of FMRP in HER2-positive breast cancer may be linked to its regulation of ECM homeostasis or specific mRNA targets that differ from its oncogenic functions in other subtypes, potentially influenced by HER2 signaling or TME interactions. This contradiction highlights the complexity of FMRP’s regulation of TME components and underscores the need for further research to elucidate the mechanisms driving its context-specific effects in cancer biology.

Building on these context-dependent roles, the mechanism by which FMRP exerts diverse regulatory functions across tumor types likely involves distinct coactivators, such as non-coding RNAs and m6A modification machinery, that modulate its RNA-binding specificity. For instance, in hepatocellular carcinoma, FMRP interacts with circZKSCAN1 to suppress WNT signaling while enhancing IL-6/STAT3 signaling, promoting metastasis [103,104]. This dual functionality highlights FMRP’s complex influence on cancer biology, setting the stage for its diverse roles in other malignancies. Transitioning to prostate cancer, FMRP’s regulatory scope expands to metabolic processes, where it collaborates with *circRBM33* to stabilize PDHA1 mRNA, thereby boosting ATP production and supporting the metabolic adaptation critical for tumor survival [105]. Additionally, in gastric cancer, it stabilizes *ITGA6* and upregulates *FZD5* mRNA via m6A binding to drive metastatic and proliferative signaling [106,107]. These findings suggest that non-coding RNAs like *circZKSCAN1* and *circRBM33* act as coactivators, directing FMRP’s target selection in a tumor-specific manner. Tumor microenvironment signals, such as cytokine-driven STAT3 activation, may further modulate these interactions, tailoring FMRP’s role in metabolic or immune regulation. Although direct evidence for these coactivator interactions remains limited, FMRP’s diverse roles across multiple cancer types highlight its potential as a therapeutic target.

## 7. Future Directions and Research Gaps

Although growing evidence connects FMRP to cellular metabolism and cancer advancement, numerous uncertainties persist. Future studies should concentrate on several critical aspects to clarify FMRP’s mechanistic contributions to cancer biology.

Detailed mechanisms of FMRP in metabolic pathways: Further studies are needed to uncover how FMRP regulates glycolysis, mitochondrial function, and lipid metabolism in different cancer types. Identifying FMRP’s downstream targets and its interaction with other metabolic regulators will provide a clearer picture of its role in tumor metabolism.

FMRP in tumor immune modulation: Understanding how FMRP modulates immune cell metabolism and contributes to immune evasion in the TME is crucial. Research focused on the interaction between FMRP and immune cells, including T cells, macrophages, and dendritic cells, will deepen our understanding of how FMRP influences tumor immunity and response to immunotherapy.

Therapeutic targeting of FMRP: Translating FMRP’s role in cancer metabolism to clinical therapies requires the development of selective inhibitors or modulators of FMRP. Research into delivery methods, specificity, and combination therapies will be essential to overcome potential challenges and maximize therapeutic efficacy.

Clinical trials and patient stratification: Clinical studies will be necessary to validate the role of FMRP in cancer progression and therapy resistance. Classifying patients according to FMRP expression levels and metabolic characteristics might reveal its value as a biomarker for therapeutic strategies.

## 8. Conclusions

The expanding collection of evidence indicates that FMRP, typically linked to neurodevelopment, is crucial in overseeing cellular metabolism within the TME. By adjusting essential metabolic processes like glycolysis, mitochondrial activity, and lipid metabolism, FMRP supports tumor expansion, immune escape, and resistance to treatment. As our understanding of FMRP’s role in cancer metabolism deepens, it opens new possibilities for therapeutic intervention. Targeting FMRP, particularly in combination with other therapies, holds promise for improving cancer treatment outcomes, especially in tumors that rely heavily on metabolic plasticity for survival.

## Data Availability

Not applicable.

## References

[1] Hagerman R.J., Berry-Kravis E., Hazlett H.C., Bailey D.B., Moine H., Kooy R.F., Tassone F., Gantois I., Sonenberg N., Mandel J.L. (2017). Fragile X syndrome. Nat. Rev. Dis. Primers.

[2] Liu X.S., Wu H., Krzisch M., Wu X., Graef J., Muffat J., Hnisz D., Li C.H., Yuan B., Xu C. (2018). Rescue of Fragile X Syndrome Neurons by DNA Methylation Editing of the *FMR1* Gene. Cell.

[3] Richter J.D., Zhao X. (2021). The molecular biology of FMRP: New insights into fragile X syndrome. Nat. Rev. Neurosci..

[4] Kurosaki T., Mitsutomi S., Hewko A., Akimitsu N., Maquat L.E. (2022). Integrative omics indicate FMRP sequesters mRNA from translation and deadenylation in human neuronal cells. Mol. Cell.

[5] Zeng Q., Saghafinia S., Chryplewicz A., Fournier N., Christe L., Xie Y.Q., Guillot J., Yucel S., Li P., Galván J.A. (2022). Aberrant hyperexpression of the RNA binding protein FMRP in tumors mediates immune evasion. Science.

[6] Yoshida G.J. (2015). Metabolic reprogramming: The emerging concept and associated therapeutic strategies. J. Exp. Clin. Cancer Res..

[7] Xia L., Oyang L., Lin J., Tan S., Han Y., Wu N., Yi P., Tang L., Pan Q., Rao S. (2021). The cancer metabolic reprogramming and immune response. Mol. Cancer.

[8] de Esch C.E., Ghazvini M., Loos F., Schelling-Kazaryan N., Widagdo W., Munshi S.T., van der Wal E., Douben H., Gunhanlar N., Kushner S.A. (2014). Epigenetic characterization of the *FMR1* promoter in induced pluripotent stem cells from human fibroblasts carrying an unmethylated full mutation. Stem Cell Rep..

[9] Elhanani O., Ben-Uri R., Keren L. (2023). Spatial profiling technologies illuminate the tumor microenvironment. Cancer Cell.

[10] Geng J., Khaket T.P., Pan J., Li W., Zhang Y., Ping Y., Cobos Sillero M.I., Lu B. (2023). Deregulation of ER-mitochondria contact formation and mitochondrial calcium homeostasis mediated by *VDAC* in fragile X syndrome. Dev. Cell.

[11] Chen X., Zhao Y., Wang D., Lin Y., Hou J., Xu X., Wu J., Zhong L., Zhou Y., Shen J. (2021). The HNF4α-BC200-*FMR1*-Positive Feedback Loop Promotes Growth and Metastasis in Invasive Mucinous Lung Adenocarcinoma. Cancer Res..

[12] Sirois C.L., Guo Y., Li M., Wolkoff N.E., Korabelnikov T., Sandoval S., Lee J., Shen M., Contractor A., Sousa A.M.M. (2024). CGG repeats in the human *FMR1* gene regulate mRNA localization and cellular stress in developing neurons. Cell Rep..

[13] Hu Y., Gao Q., Ma S., Yu P., Ding S., Yao X., Zhang Z., Lu S., Lu M., Zhang J. (2022). *FMR1* promotes the progression of colorectal cancer cell by stabilizing EGFR mRNA in an m(6)A-dependent manner. Cell Death Dis..

[14] Lucá R., Averna M., Zalfa F., Vecchi M., Bianchi F., La Fata G., Del Nonno F., Nardacci R., Bianchi M., Nuciforo P. (2013). The fragile X protein binds mRNAs involved in cancer progression and modulates metastasis formation. EMBO Mol. Med..

[15] Taha M.S., Haghighi F., Stefanski A., Nakhaei-Rad S., Kazemein Jasemi N.S., Al Kabbani M.A., Görg B., Fujii M., Lang P.A., Häussinger D. (2021). Novel FMRP interaction networks linked to cellular stress. Febs J..

[16] Baldi S., Amer B., Alnadari F., Al-Mogahed M., Gao Y., Gamallat Y. (2024). The Prognostic and Therapeutic Potential of Fragile X Mental Retardation 1 (*FMR1*) Gene Expression in Prostate Adenocarcinoma: Insights into Survival Outcomes and Oncogenic Pathway Modulation. Int. J. Mol. Sci..

[17] Casingal C.R., Kikkawa T., Inada H., Sasaki Y., Osumi N. (2020). Identification of FMRP target mRNAs in the developmental brain: FMRP might coordinate Ras/MAPK, Wnt/β-catenin, and mTOR signaling during corticogenesis. Mol. Brain.

[18] Hua H., Kong Q., Zhang H., Wang J., Luo T., Jiang Y. (2019). Targeting mTOR for cancer therapy. J. Hematol. Oncol..

[19] Mossmann D., Park S., Hall M.N. (2018). mTOR signalling and cellular metabolism are mutual determinants in cancer. Nat. Rev. Cancer.

[20] Yang H., Wang Y., Xiang Y., Yadav T., Ouyang J., Phoon L., Zhu X., Shi Y., Zou L., Lan L. (2022). FMRP promotes transcription-coupled homologous recombination via facilitating *TET1*-mediated m5C RNA modification demethylation. Proc. Natl. Acad. Sci. USA.

[21] DeBerardinis R.J., Lum J.J., Hatzivassiliou G., Thompson C.B. (2008). The biology of cancer: Metabolic reprogramming fuels cell growth and proliferation. Cell Metab..

[22] Leboucher A., Pisani D.F., Martinez-Gili L., Chilloux J., Bermudez-Martin P., Van Dijck A., Ganief T., Macek B., Becker J.A.J., Le Merrer J. (2019). The translational regulator FMRP controls lipid and glucose metabolism in mice and humans. Mol. Metab..

[23] Tabet R., Vitale N., Moine H. (2016). Fragile X syndrome: Are signaling lipids the missing culprits?. Biochimie.

[24] Gill K.S., Fernandes P., O’Donovan T.R., McKenna S.L., Doddakula K.K., Power D.G., Soden D.M., Forde P.F. (2016). Glycolysis inhibition as a cancer treatment and its role in an anti-tumour immune response. Biochim. Biophys. Acta.

[25] Fernie A.R., Carrari F., Sweetlove L.J. (2004). Respiratory metabolism: Glycolysis, the TCA cycle and mitochondrial electron transport. Curr. Opin. Plant Biol..

[26] Liberti M.V., Locasale J.W. (2016). The Warburg Effect: How Does it Benefit Cancer Cells?. Trends Biochem. Sci..

[27] Vaupel P., Schmidberger H., Mayer A. (2019). The Warburg effect: Essential part of metabolic reprogramming and central contributor to cancer progression. Int. J. Radiat. Biol..

[28] Licznerski P., Park H.A., Rolyan H., Chen R., Mnatsakanyan N., Miranda P., Graham M., Wu J., Cruz-Reyes N., Mehta N. (2020). ATP Synthase c-Subunit Leak Causes Aberrant Cellular Metabolism in Fragile X Syndrome. Cell.

[29] Ryu K.W., Fung T.S., Baker D.C., Saoi M., Park J., Febres-Aldana C.A., Aly R.G., Cui R., Sharma A., Fu Y. (2024). Cellular ATP demand creates metabolically distinct subpopulations of mitochondria. Nature.

[30] Fenton A.R., Peng R., Bond C., Hugelier S., Lakadamyali M., Chang Y.W., Holzbaur E.L.F., Jongens T.A. (2024). FMRP regulates MFF translation to locally direct mitochondrial fission in neurons. Nat. Cell Biol..

[31] Sirois C.L., Sandoval S.O., Zhao X. (2024). FMRP gains mitochondrial fission control. Nat. Cell Biol..

[32] Zhang R., Peng X., Du J.X., Boohaker R., Estevao I.L., Grajeda B.I., Cox M.B., Almeida I.C., Lu W. (2023). Oncogenic KRASG12D Reprograms Lipid Metabolism by Upregulating SLC25A1 to Drive Pancreatic Tumorigenesis. Cancer Res..

[33] Zhang Y., Wu M.J., Lu W.C., Li Y.C., Chang C.J., Yang J.Y. (2024). Metabolic switch regulates lineage plasticity and induces synthetic lethality in triple-negative breast cancer. Cell Metab..

[34] Zhu L., Zhu X., Wu Y. (2022). Effects of Glucose Metabolism, Lipid Metabolism, and Glutamine Metabolism on Tumor Microenvironment and Clinical Implications. Biomolecules.

[35] Jiang S., Li H., Zhang L., Mu W., Zhang Y., Chen T., Wu J., Tang H., Zheng S., Liu Y. (2024). Generic Diagramming Platform (GDP): A comprehensive database of high-quality biomedical graphics. Nucleic Acids Res..

[36] Pieretti M., Zhang F.P., Fu Y.H., Warren S.T., Oostra B.A., Caskey C.T., Nelson D.L. (1991). Absence of expression of the FMR-1 gene in fragile X syndrome. Cell.

[37] Shah S., Sharp K.J., Raju Ponny S., Lee J., Watts J.K., Berry-Kravis E., Richter J.D. (2023). Antisense oligonucleotide rescue of CGG expansion-dependent *FMR1* mis-splicing in fragile X syndrome restores FMRP. Proc. Natl. Acad. Sci. USA.

[38] Musco G., Stier G., Joseph C., Castiglione Morelli M.A., Nilges M., Gibson T.J., Pastore A. (1996). Three-dimensional structure and stability of the KH domain: Molecular insights into the fragile X syndrome. Cell.

[39] Krueger D.D., Bear M.F. (2011). Toward fulfilling the promise of molecular medicine in fragile X syndrome. Annu. Rev. Med..

[40] Napoli E., Ross-Inta C., Song G., Wong S., Hagerman R., Gane L.W., Smilowitz J.T., Tassone F., Giulivi C. (2016). Premutation in the Fragile X Mental Retardation 1 (*FMR1*) Gene Affects Maternal Zn-milk and Perinatal Brain Bioenergetics and Scaffolding. Front. Neurosci..

[41] Bassell G.J., Warren S.T. (2008). Fragile X syndrome: Loss of local mRNA regulation alters synaptic development and function. Neuron.

[42] Higashimori H., Schin C.S., Chiang M.S., Morel L., Shoneye T.A., Nelson D.L., Yang Y. (2016). Selective Deletion of Astroglial FMRP Dysregulates Glutamate Transporter GLT1 and Contributes to Fragile X Syndrome Phenotypes In Vivo. J. Neurosci..

[43] Pedini G., Buccarelli M., Bianchi F., Pacini L., Cencelli G., D’Alessandris Q.G., Martini M., Giannetti S., Sasso F., Melocchi V. (2022). FMRP modulates the Wnt signalling pathway in glioblastoma. Cell Death Dis..

[44] Bejarano L., Jordāo M.J.C., Joyce J.A. (2021). Therapeutic Targeting of the Tumor Microenvironment. Cancer Discov..

[45] Bilotta M.T., Antignani A., Fitzgerald D.J. (2022). Managing the TME to improve the efficacy of cancer therapy. Front. Immunol..

[46] Hanahan D., Monje M. (2023). Cancer hallmarks intersect with neuroscience in the tumor microenvironment. Cancer Cell.

[47] Lee H.W., Xu Y., He L., Choi W., Gonzalez D., Jin S.W., Simons M. (2021). Role of Venous Endothelial Cells in Developmental and Pathologic Angiogenesis. Circulation.

[48] Leung S.W.S., Shi Y. (2022). The glycolytic process in endothelial cells and its implications. Acta Pharmacol. Sin..

[49] Zhao X., Wang Y., Meng C., Fang N. (2018). FMRP regulates endothelial cell proliferation and angiogenesis via the miR-181a-CaM-CaMKII pathway. Cell Biol. Int..

[50] Ma Y.X., Lei C., Ye T., Wan Q.Q., Wang K.Y., Zhu Y.N., Li L., Liu X.F., Niu L.Z., Tay F.R. (2025). Silicon Enhances Functional Mitochondrial Transfer to Improve Neurovascularization in Diabetic Bone Regeneration. Adv. Sci..

[51] Cao J., Yan Q. (2020). Cancer Epigenetics, Tumor Immunity, and Immunotherapy. Trends Cancer.

[52] Saravia J., Raynor J.L., Chapman N.M., Lim S.A., Chi H. (2020). Signaling networks in immunometabolism. Cell Res..

[53] St Paul M., Ohashi P.S. (2020). The Roles of CD8(+) T Cell Subsets in Antitumor Immunity. Trends Cell Biol..

[54] Reina-Campos M., Scharping N.E., Goldrath A.W. (2021). CD8(+) T cell metabolism in infection and cancer. Nat. Rev. Immunol..

[55] Cao J., Liao S., Zeng F., Liao Q., Luo G., Zhou Y. (2023). Effects of altered glycolysis levels on CD8(+) T cell activation and function. Cell Death Dis..

[56] Geltink R.I.K., Kyle R.L., Pearce E.L. (2018). Unraveling the Complex Interplay Between T Cell Metabolism and Function. Annu. Rev. Immunol..

[57] Nayak T., Trotter J., Sakry D. (2018). The Intracellular Cleavage Product of the NG2 Proteoglycan Modulates Translation and Cell-Cycle Kinetics via Effects on mTORC1/FMRP Signaling. Front. Cell Neurosci..

[58] Szwed A., Kim E., Jacinto E. (2021). Regulation and metabolic functions of mTORC1 and mTORC2. Physiol. Rev..

[59] Hooshmandi M., Sharma V., Thörn Perez C., Sood R., Krimbacher K., Wong C., Lister K.C., Ureña Guzmán A., Bartley T.D., Rocha C. (2023). Excitatory neuron-specific suppression of the integrated stress response contributes to autism-related phenotypes in fragile X syndrome. Neuron.

[60] Vitale I., Manic G., Coussens L.M., Kroemer G., Galluzzi L. (2019). Macrophages and Metabolism in the Tumor Microenvironment. Cell Metab..

[61] Boutilier A.J., Elsawa S.F. (2021). Macrophage Polarization States in the Tumor Microenvironment. Int. J. Mol. Sci..

[62] Liu P.S., Wang H., Li X., Chao T., Teav T., Christen S., Di Conza G., Cheng W.C., Chou C.H., Vavakova M. (2017). α-ketoglutarate orchestrates macrophage activation through metabolic and epigenetic reprogramming. Nat. Immunol..

[63] Mehla K., Singh P.K. (2019). Metabolic Regulation of Macrophage Polarization in Cancer. Trends Cancer.

[64] Su P., Wang Q., Bi E., Ma X., Liu L., Yang M., Qian J., Yi Q. (2020). Enhanced Lipid Accumulation and Metabolism Are Required for the Differentiation and Activation of Tumor-Associated Macrophages. Cancer Res..

[65] Wu K.K., Xu X., Wu M., Li X., Hoque M., Li G.H.Y., Lian Q., Long K., Zhou T., Piao H. (2024). MDM2 induces pro-inflammatory and glycolytic responses in M1 macrophages by integrating iNOS-nitric oxide and HIF-1α pathways in mice. Nat. Commun..

[66] Zhang D., Tang Z., Huang H., Zhou G., Cui C., Weng Y., Liu W., Kim S., Lee S., Perez-Neut M. (2019). Metabolic regulation of gene expression by histone lactylation. Nature.

[67] Haschemi A., Kosma P., Gille L., Evans C.R., Burant C.F., Starkl P., Knapp B., Haas R., Schmid J.A., Jandl C. (2012). The sedoheptulose kinase *CARKL* directs macrophage polarization through control of glucose metabolism. Cell Metab..

[68] Beatty G.L., Gladney W.L. (2015). Immune escape mechanisms as a guide for cancer immunotherapy. Clin. Cancer Res..

[69] Kao K.C., Vilbois S., Tsai C.H., Ho P.C. (2022). Metabolic communication in the tumour-immune microenvironment. Nat. Cell Biol..

[70] Hatzioannou A., Banos A., Sakelaropoulos T., Fedonidis C., Vidali M.S., Köhne M., Händler K., Boon L., Henriques A., Koliaraki V. (2020). An intrinsic role of IL-33 in T(reg) cell-mediated tumor immunoevasion. Nat. Immunol..

[71] Liu Y., Cai Y., Liu L., Wu Y., Xiong X. (2018). Crucial biological functions of CCL7 in cancer. PeerJ.

[72] Maurin T., Zongaro S., Bardoni B. (2014). Fragile X Syndrome: From molecular pathology to therapy. Neurosci. Biobehav. Rev..

[73] Zhang B., Zhang J., Chen H., Qiao D., Guo F., Hu X., Qin C., Jin X., Zhang K., Wang C. (2024). Role of FMRP in AKT/mTOR pathway-mediated hippocampal autophagy in fragile X syndrome. Prog. Neuropsychopharmacol. Biol. Psychiatry.

[74] Wang N., Shi B., Man X., Wu W., Cao J. (2024). [High expression of fragile X mental retardation protein inhibits ferroptosis of colorectal tumor cells by activating the RAS/MAPK signaling pathway]. Nan Fang. Yi Ke Da Xue Xue Bao.

[75] Zhang Y., Zhang Z. (2020). The history and advances in cancer immunotherapy: Understanding the characteristics of tumor-infiltrating immune cells and their therapeutic implications. Cell Mol. Immunol..

[76] Gill J., Prasad V. (2019). A reality check of the accelerated approval of immune-checkpoint inhibitors. Nat. Rev. Clin. Oncol..

[77] Bagchi S., Yuan R., Engleman E.G. (2021). Immune Checkpoint Inhibitors for the Treatment of Cancer: Clinical Impact and Mechanisms of Response and Resistance. Annu. Rev. Pathol..

[78] Jia Y., Jia R., Chen Y., Lin X., Aishan N., Li H., Wang L., Zhang X., Ruan J. (2025). The role of RNA binding proteins in cancer biology: A focus on FMRP. Genes Dis..

[79] Peng R., Huang Q., Wang L., Qiao G., Huang X., Jiang J., Chu X. (2024). G-Quadruplex RNA Based PROTAC Enables Targeted Degradation of RNA Binding Protein FMRP for Tumor Immunotherapy. Angew. Chem. Int. Ed. Engl..

[80] Szeto G.L., Finley S.D. (2019). Integrative Approaches to Cancer Immunotherapy. Trends Cancer.

[81] Bader J.E., Voss K., Rathmell J.C. (2020). Targeting Metabolism to Improve the Tumor Microenvironment for Cancer Immunotherapy. Mol. Cell.

[82] Granchi C. (2018). ATP citrate lyase (ACLY) inhibitors: An anti-cancer strategy at the crossroads of glucose and lipid metabolism. Eur. J. Med. Chem..

[83] Gao M., Huang J., Jiang X., Yuan Y., Pang H., Luo S., Wang N., Yao C., Lin Z., Pu D. (2020). Regulation of aerobic glycolysis to decelerate tumor proliferation by small molecule inhibitors targeting glucose transporters. Protein Cell.

[84] Walcher L., Kistenmacher A.K., Suo H., Kitte R., Dluczek S., Strauß A., Blaudszun A.R., Yevsa T., Fricke S., Kossatz-Boehlert U. (2020). Cancer Stem Cells-Origins and Biomarkers: Perspectives for Targeted Personalized Therapies. Front. Immunol..

[85] Yang L., Shi P., Zhao G., Xu J., Peng W., Zhang J., Zhang G., Wang X., Dong Z., Chen F. (2020). Targeting cancer stem cell pathways for cancer therapy. Signal Transduct. Target. Ther..

[86] Hagerman P.J., Hagerman R. (2021). Fragile X syndrome. Curr. Biol..

[87] Hu Y., Nie W., Lyu L., Zhang X., Wang W., Zhang Y., He S., Guo A., Liu F., Wang B. (2024). Tumor-Microenvironment-Activatable Nanoparticle Mediating Immunogene Therapy and M2 Macrophage-Targeted Inhibitor for Synergistic Cancer Immunotherapy. ACS Nano.

[88] Lim Z.F., Ma P.C. (2019). Emerging insights of tumor heterogeneity and drug resistance mechanisms in lung cancer targeted therapy. J. Hematol. Oncol..

[89] Wei G., Wang Y., Yang G., Wang Y., Ju R. (2021). Recent progress in nanomedicine for enhanced cancer chemotherapy. Theranostics.

[90] Lumaban J.G., Nelson D.L. (2015). The Fragile X proteins Fmrp and Fxr2p cooperate to regulate glucose metabolism in mice. Hum. Mol. Genet..

[91] Huang M., Liu M., Wang R., Man Y., Zhou H., Xu Z.X., Wang Y. (2024). The crosstalk between glucose metabolism and telomerase regulation in cancer. Biomed. Pharmacother..

[92] Habbas K., Cakil O., Zámbó B., Tabet R., Riet F., Dembele D., Mandel J.L., Hocquemiller M., Laufer R., Piguet F. (2022). AAV-delivered diacylglycerol kinase DGKk achieves long-term rescue of fragile X syndrome mouse model. EMBO Mol. Med..

[93] Zafarullah M., Palczewski G., Rivera S.M., Hessl D.R., Tassone F. (2020). Metabolic profiling reveals dysregulated lipid metabolism and potential biomarkers associated with the development and progression of Fragile X-Associated Tremor/Ataxia Syndrome (FXTAS). Faseb J..

[94] Zhang Y., Wu T., Han J. (2023). Targeting FMRP: A new window for cancer immunotherapy. MedComm (2020).

[95] Wang N., Shi B., Ding L., Zhang X., Ma X., Guo S., Qiao X., Wang L., Ma D., Cao J. (2024). FMRP protects breast cancer cells from ferroptosis by promoting *SLC7A11* alternative splicing through interacting with hnRNPM. Redox Biol..

[96] Zhang D., Xu X., Ye Q. (2021). Metabolism and immunity in breast cancer. Front. Med..

[97] Liu Y., Yu K., Zhang K., Niu M., Chen Q., Liu Y., Wang L., Zhang N., Li W., Zhong X. (2023). O-GlcNAcylation promotes topoisomerase IIα catalytic activity in breast cancer chemoresistance. EMBO Rep..

[98] Zhang Z., Li X., Yang F., Chen C., Liu P., Ren Y., Sun P., Wang Z., You Y., Zeng Y.X. (2021). DHHC9-mediated GLUT1 S-palmitoylation promotes glioblastoma glycolysis and tumorigenesis. Nat. Commun..

[99] Guo D., Tong Y., Jiang X., Meng Y., Jiang H., Du L., Wu Q., Li S., Luo S., Li M. (2022). Aerobic glycolysis promotes tumor immune evasion by hexokinase2-mediated phosphorylation of IκBα. Cell Metab..

[100] Carotti S., Zingariello M., Francesconi M., D’Andrea L., Latasa M.U., Colyn L., Fernandez-Barrena M.G., Flammia R.S., Falchi M., Righi D. (2021). Fragile X mental retardation protein in intrahepatic cholangiocarcinoma: Regulating the cancer cell behavior plasticity at the leading edge. Oncogene.

[101] Rahnemai-Azar A.A., Weisbrod A., Dillhoff M., Schmidt C., Pawlik T.M. (2017). Intrahepatic cholangiocarcinoma: Molecular markers for diagnosis and prognosis. Surg. Oncol..

[102] Caredda E., Pedini G., D’Amico F., Scioli M.G., Pacini L., Orsaria P., Vanni G., Buonomo O.C., Orlandi A., Bagni C. (2023). FMRP expression in primary breast tumor cells correlates with recurrence and specific site of metastasis. PLoS ONE.

[103] Shen Z., Liu B., Wu B., Zhou H., Wang X., Cao J., Jiang M., Zhou Y., Guo F., Xue C. (2021). FMRP regulates STAT3 mRNA localization to cellular protrusions and local translation to promote hepatocellular carcinoma metastasis. Commun. Biol..

[104] Zhu Y.J., Zheng B., Luo G.J., Ma X.K., Lu X.Y., Lin X.M., Yang S., Zhao Q., Wu T., Li Z.X. (2019). Circular RNAs negatively regulate cancer stem cells by physically binding FMRP against CCAR1 complex in hepatocellular carcinoma. Theranostics.

[105] Zhong C., Long Z., Yang T., Wang S., Zhong W., Hu F., Teoh J.Y., Lu J., Mao X. (2023). M6A-modified *circRBM33* promotes prostate cancer progression via PDHA1-mediated mitochondrial respiration regulation and presents a potential target for ARSI therapy. Int. J. Biol. Sci..

[106] Wang X., Xu K., Liao X., Rao J., Huang K., Gao J., Xu G., Wang D. (2022). Construction of a survival nomogram for gastric cancer based on the cancer genome atlas of m6A-related genes. Front. Genet..

[107] Yang J., Wu Z., Wu X., Chen S., Xia X., Zeng J. (2022). Constructing and validating of m6a-related genes prognostic signature for stomach adenocarcinoma and immune infiltration: Potential biomarkers for predicting the overall survival. Front. Oncol..

